# Intra-individual reliability of blood bicarbonate responses and gastrointestinal symptoms following sodium citrate supplementation

**DOI:** 10.1080/15502783.2026.2629830

**Published:** 2026-02-09

**Authors:** Chris J. McManus, Bernard X.W. Liew, Sally P.W. Waterworth, Henry C. Chung

**Affiliations:** a School of Sport, Rehabilitation and Exercise Sciences, University of Essex, Colchester, Essex, United Kingdom

**Keywords:** Sodium citrate, blood bicarbonate, extracellular buffering, reliability, gastrointestinal symptoms, ergogenic aids

## Abstract

**Background:**

Sodium citrate (SC) can elevate extracellular buffering capacity, yet the intra-individual reliability of its blood bicarbonate ([HCO₃^−^]) kinetics and gastrointestinal (GI) responses is unclear, limiting individualized dosing strategies.

**Methods:**

Twelve healthy males (21 ± 1 yr) ingested a solution containing 0.5 g·kg^−1^ SC on two visits 3–7 days apart. Capillary [HCO₃^−^] was sampled at baseline and every 30 min to 240 min to derive baseline and peak [HCO₃^−^], time to peak (TTP), time to exceed +5 and +6 mmol·L^−1^ above baseline, and area under the curve (AUC). Reliability was quantified with ICC, typical error (TE), and CV; a Monte Carlo simulation estimated the probability of exceeding +5 and +6 mmol·L^−1^ at each time point. GI symptoms (12-item questionnaire) were recorded concurrently.

**Results:**

[HCO₃^−^] rose significantly over time from 30 min in both visits (*p* < 0.001). Reliability was moderate for baseline [HCO₃^−^] (ICC = 0.72 [0.25, 0.91]; CV = 3.5%) and AUC (ICC = 0.56; CV = 3.5%), but poor for peak [HCO₃^−^] (ICC = 0.23 [−0.29, 0.68]; CV = 5.4%) and all time-based metrics, including TTP (ICC = 0.07; TE = 49.1 min; CV = 32.5%) and time to +5 and +6 mmol·L^−1^. Simulation showed an ≥ 80% probability of exceeding +5 mmol·L^−1^ from 120–240 min (83.9–85.8%), whereas +6 mmol·L^−1^ peaked at 69.7% (150 min). GI symptoms were common, unchanged across visits, and moderately reliable for overall burden (ICC = 0.61; TE = 2.63; CV = 46.6%).

**Conclusion:**

SC elicits a consistent group-level alkalosis, yet individual timing metrics are unreliable. Concentration-based indices are more stable for monitoring. Practically, a 2–3 h ingestion window maximizes the probability of achieving ≥+5 mmol·L^−1^, but individual profiling is recommended where precise timing is critical.

## 1 Introduction

Exercise-induced metabolic acidosis represents a primary limiting factor in high-intensity exercise performance, characterised by intramuscular and systemic hydrogen ion (H^+^) accumulation [1,2]. During maximal exercise, intracellular pH decreases from approximately 7.1 to 6.5, primarily due to anaerobic glycolysis and subsequent lactate production with concomitant H^+^ release, increasing the internal acidity [2,3]. This acidification impairs key glycolytic enzymes, disrupts excitation-contraction coupling, and ultimately compromises exercise capacity (1).

The body's endogenous buffering systems, including intracellular phosphates and proteins alongside extracellular bicarbonate [HCO₃^−^], provide the primary defence against exercise-induced acidosis [1,2]. However, these systems become saturated during exercise intensities exceeding 90% V̇O₂max, prompting investigation into exogenous buffering strategies [1]. Alkalinising agents, particularly sodium bicarbonate (NaHCO₃) and sodium citrate (SC), have emerged as potential ergogenic aids to enhance buffering capacity and delay fatigue onset [2,4–6].

Supplementing with SC can potentially delay fatigue by increasing extracellular buffering capacity and facilitating the removal of H^+^ from muscles [1,5]. Upon ingestion, SC rapidly dissociates into sodium (Na^+^) and citrate (C_6_H_5_O_7_^−3^)ions. The citrate ion, as the conjugate base of citric acid, can accept hydrogen ions (H^+^), effectively removing free H^+^ from body fluids. Additionally, the citrate is metabolised to bicarbonate in the liver, which further contributes to buffering by increasing [HCO₃^−^] [7]. This combined process lowers the concentration of H^+^ and raises blood [HCO₃^−^], inducing a metabolic alkalosis [8]. In turn, the elevated extracellular bicarbonate and pH enhance the gradient for H^+^ efflux from working muscle, augmenting the body’s capacity to counteract exercise-induced acidosis.

Unlike NaHCO₃, citrate carries a higher negative charge and can permeate the sarcolemma, entering muscle cells and participating in metabolic processes [1]. Within muscle fibres, citrate acts as a metabolic intermediate in the Krebs cycle and plays a role in transporting acetyl-CoA to the cytosol for fatty acid synthesis [8]. It also acts as a negative allosteric effector of phosphofructokinase (PFK), potentially interfering with glycolysis by inhibiting PFK activity [8]. However, this inhibition may be offset during prolonged high-intensity exercise where lactate production peaks [9].

The ergogenic benefits of sodium citrate supplementation remain equivocal. Performance benefits are more consistently observed in short-duration, high-intensity cycling efforts lasting approximately 1 to 4 minutes, with improvements in work output and peak power particularly evident within this range [10–12]. By contrast, supplementation appears less effective during very short bouts (<60 seconds) [12] or prolonged endurance exercise (>7 minutes) [13], where physiological demands and buffering requirements may differ. Variability in findings across studies may also reflect inter-individual differences, small sample sizes, methodological differences, and environmental influences [10].

An important consideration in SC supplementation is achieving a sufficient increase in blood [HCO₃^−^] to elicit ergogenic effects. Heibel et al. suggested that any increase in blood [HCO₃^−^] could enhance buffering capacity, with a rise of + 5 mmol·L^−1^ necessary for potential performance benefits and + 6 mmol·L^−1^ producing 'almost certain ergogenic effects' [14,15]. However, recent meta-analyses indicate that exceeding a + 6 mmol·L^−1^ increase does not necessarily confer additional performance advantages over increases between + 4 and + 6 mmol·L^−1^ [16]. This suggests that achieving a rise above the minimal threshold is crucial, but further increases may not yield proportional benefits.

Dosage and timing of SC ingestion are critical for optimising its ergogenic potential while minimising gastrointestinal (GI) symptoms [14,15]. Early studies using low doses of SC (e.g. 5 g) failed to show significant effects on performance, likely due to insufficient increases in blood [HCO₃^−^] [1]. Subsequent research demonstrated a linear increase in blood [HCO₃^−^]with doses ranging from 0.1 to 0.5 g·kg^−1^·body mass (BM), with 0.5 g·kg^−1^·BM appearing to provide performance benefits [10]. However, higher doses can lead to increased GI discomfort and negatively affect performance outcomes [10,17].

The time to peak (TTP) in blood [HCO₃^−^] levels varies among individuals, with studies reporting TTP ranging from 90 minutes to over 200 minutes post-ingestion [10,18]. Urwin et al. found significant increases in blood [HCO₃^−^] at 90 minutes post-ingestion across different doses, with TTP occurring at 180 to 215 minutes for all dosages [18]. Different modes of administration, such as soluble solutions or gel capsules, can influence absorption rates and GI symptoms. While capsules may delay TTP, they do not necessarily reduce GI discomfort compared to soluble solutions [17,19,20].

Inter-individual variability in the pharmacokinetic responses to SC supplementation presents a challenge in establishing standardised protocols for athletes [1,14]. Studies have documented variations in TTP, peak blood [HCO₃^−^] levels, and the duration over which [HCO₃^−^] levels remain above the performance-enhancing threshold [1,17,18]. Only one study has investigated intra-individual variation in blood acid–base responses following NaHCO₃ supplementation, revealing consistency in time spent above the + 5 mmol·L^−1^ threshold among participants [6]. However, no equivalent studies have been conducted for SC supplementation.

Understanding the individual reliability of blood [HCO₃^−^] and pH responses to SC supplementation is crucial for tailoring strategies to optimise performance benefits when using ergogenic aids. Athletes need precise timing to schedule their intake to coincide with peak buffering capacity while minimising the risk of GI discomfort during competition. Although no study with sodium citrate has yet paired an individualised ingestion strategy with a performance test, individualised sodium bicarbonate protocols timed to each athlete’s peak alkalosis have yielded ~0.7–2.6% performance gains versus placebo or control across running and cycling time trials [21–24]. The lack of research on intra-individual variability and test–retest reliability of SC supplementation highlights a significant gap in the literature and potentially hinders maximal potential in competition.

The primary aim of this research is to investigate the individual reliability of TTP blood bicarbonate following sodium citrate supplementation. We seek to determine the time course of changes in blood [HCO₃^−^] over four hours post-ingestion of SC, to identify potential individual differences. We hypothesise that there will be no significant difference in blood [HCO₃^−^] and TTP when comparing mean data between test sessions. Additionally, we expect the time courses for blood [HCO₃^−^], time to surpass, and time spent over the performance-enhancing threshold of + 5 mmol·L^−1^ to be consistent across sessions. A secondary objective was to investigate the reliability of GI symptom responses to SC ingestion, enabling evaluation of the consistency of any discomfort reported across repeated trials.

## 2 Materials and methods

### 2.1 Participants

Twelve healthy male participants (Table 1) were recruited via printed advertisements, social media platforms, and snowball sampling. The sample size was determined *a priori* based on established recommendations for estimating intraclass correlation coefficients with adequate confidence interval precision. For studies aiming to differentiate moderate from poor reliability (e.g. ICC ≈ 0.6 vs 0.2), a sample of 10–15 participants with two observations is typically sufficient [25,26]. All participants were free from chronic diseases or conditions known to impact metabolic responses. Ethical approval was obtained from the University of Essex Ethics Subcommittee (ETH2324-0173). Participants provided written informed consent after completing comprehensive health screening procedures, including verbal confirmation of eligibility criteria.

**Table 1.** Descriptive characteristics of participants.

	Mean ± SD
Age (years)	21 ± 1
Body mass (kg)	82.4 ± 9.1
Stature (m)	1.81 ± 0.05
Volume of SC consumed (g)	41.2 ± 4.6

SC; sodium citrate.

### 2.2 Pre-experimental procedures

Participants attended two in-person visits to the university's sport and exercise laboratory, where the environment is controlled and maintained at a temperature of 20 °C, humidity at 50%, barometric pressure at standard sea level of 1013 hPa, and remained consistent between visits. Visits were separated by 3–7 days to ensure sufficient washout of blood bicarbonate [HCO₃^−^] levels to return to baseline levels (7). All participants refrained from strenuous exercise, caffeine, and alcohol for at least 24 hours before each visit to avoid confounding metabolic influences. Additionally, participants were instructed to replicate their dietary intake in the 24 hours before each visit. Compliance was assessed using a validated online 24-hour dietary recall method (Intake24) [27], and total energy intake (kcal),macronutrient composition and sodium intake were compared between visit 1 and visit 2 to evaluate dietary standardisation. In addition, participants were instructed to consume their habitual breakfast or light meal approximately 2 hours prior to arrival. At visit 1, the timing and composition of this meal were verbally recorded and reinforced to ensure replication before visit 2, with verbal confirmation of compliance obtained upon arrival.

### 2.3 Experimental design

Anthropometric measurements were collected using a SECA 213 Portable Stadiometer (height) and SECA 813 Electronic Scales (body mass). Participants consumed a sodium citrate (SC) solution consisting of 0.5 g·kg^−1^·body mass (BM) SC powder (Special Ingredients® Sodium Citrate Acidity Regulator, food-grade) (see Table 1), mixed with 300 mL of water and 45 mL of orange-flavoured squash (Capri-Sun Fruit Squash, no-added-sugar concentrate, diluted). The squash contributed only a minimal amount of carbohydrate (~1–2 g of naturally occurring sugars in 45 mL) and no added electrolytes. Its inclusion was to improve the taste and acceptability of the solution. To aid gastrointestinal tolerance and absorption, the solution was ingested slowly in small sips over ≤ 10 minutes rather than as a single bolus. The chosen dosage aligns with previously established recommendations to minimise gastrointestinal (GI) discomfort while ensuring ergogenic efficacy [28]. No separate placebo condition was included in this study. Our primary aim was to assess the intra-individual reliability of responses to sodium citrate itself, using a repeated-measures design in which each participant served as their own control across two identical supplementation trials. This design maximises sensitivity to the consistency of the SC-induced response by reducing between-subject variability. Adding a placebo trial for every participant was deemed impractical for this reliability-focused investigation.

Blood samples were obtained using capillary finger-prick sampling (Owen Mumford Unistik 3 Normal Lancets) at baseline (pre-ingestion) and 30-minute intervals for 240 minutes post-ingestion, yielding nine samples per participant. Blood was collected into lithium-heparin-coated Minivette® Point-of-Care test (POCT) capillary tubes. Participants remained seated in the laboratory throughout testing, abstaining from food intake. Water consumption was permitted ad libitum (unrestricted and not recorded) during the 4-hour post-ingestion period.

### 2.4 Blood bicarbonate [HCO₃^−^] analysis

Blood [HCO₃^−^] was determined using the iSTAT 1 Analyser (Abbott Point-of-Care, East Windsor, NJ) with CG4+ cartridges. Within two minutes of sampling, 85 µL of blood was transferred into the cartridge and immediately analysed. The iSTAT device has been validated for clinical and sport science use, providing accurate and reliable measurements of acid–base variables (including [HCO₃^−^]) across a range of conditions [29]. For example, Dascombe et al. reported strong intraclass correlation for iSTAT bicarbonate readings (ICC = 0.81 at rest) using the same CG4 + cartridge type [29]. In our study, the primary outcomes from the blood analysis included peak blood [HCO₃^−^] concentration, time to peak (TTP), and the change in blood HCO₃^−^ from baseline (ΔHCO₃^−^). Additionally, the initial time point at which the increase in blood [HCO₃^−^] exceeded 5 mmol·L^−1^ above baseline (Δ5 mmol·L^−1^) and the duration sustained above this threshold were calculated to characterise individual responses to SC supplementation. All equipment was calibrated to the manufacturer's specifications before every trial.

### 2.5 Gastrointestinal discomfort

Gastrointestinal discomfort was assessed using a validated questionnaire (Appendix A - adapted from Carr et al., 2011 [15]). Participants rated 12 GI symptoms on a Likert scale (1 = “no problem at all” to 10 = “worst it has ever been”). To standardise the scoring range, each response was corrected by subtracting 1, resulting in a 0–9 scale for each symptom. The 12 symptoms included questions relating to: reflux, heartburn, bloating, upper abdominal cramps, vomiting, nausea, intestinal cramps, flatulence, urge to defecate, loose stool, dizziness, and headache. Ratings were collected at baseline and every 30 minutes during the 240-minute monitoring period. A composite GI discomfort score was calculated by summing the adjusted scores for all 12 symptoms at each timepoint, then summing these values across all timepoints per visit, yielding a total possible score range of 0–108. No missing responses were recorded.

### 2.6 Statistical analysis

Data are reported as mean ± standard deviation unless stated otherwise. Normality of the differences between visits for each dietary variable was assessed using the Shapiro–Wilk test, which confirmed that all variables met the assumption of normality (*p* > 0.05). Consequently, paired samples *t*-tests were conducted to compare energy and macronutrient intakes between Visit 1 and Visit 2.

For each participant and visit, blood bicarbonate time series were interpolated onto a one-minute grid (0–240 minutes) using piecewise cubic Hermite interpolation (pchip). This method preserves the shape and monotonicity of the original data, enabling smooth, physiologically realistic curves without overshooting between knots. No extrapolation beyond 240 minutes was performed. Baseline was defined as the observed value at 0 minutes. From the one-minute interpolated series, we derived six metrics: baseline, absolute peak concentration (C_max_), time to peak (TTP), time to achieve a +5 mmol·L^−1^ and +6 mmol·L^−1^ increase from baseline, and area under the concentration–time curve (AUC). C_max_ and TTP were identified as the global maximum of the interpolated profile within 0–240 minutes inclusive. When multiple consecutive minutes shared the maximum value, the earliest time on that plateau was recorded. Threshold crossing times were the earliest minute at which the interpolated concentration met or exceeded baseline +5 or +6 mmol·L^−1^. If a threshold was not reached by 240 minutes, the corresponding time was recorded as missing. AUC was computed on the interpolated series over 0–240 minutes using trapezoidal integration. All times are therefore reported to the nearest minute. Because endpoints are included, peaks can occur at 0 or 240 minutes if the profile is highest at the boundary. Missing data were handled via listwise deletion for any metric where a threshold was not achieved (i.e. time to achieve a + 5 or + 6 mmol·L^−1^ increase from baseline).

Linear mixed-effects models (LME) were used to evaluate changes in both bicarbonate and GI composite scores, with Visit, Time, and their interaction included as fixed effects, and Participant as a random effect. A Type 3 ANOVA was used to assess main and interaction effects. Within-visit comparisons from baseline were assessed using repeated-measures ANOVA with Tukey–Kramer post hoc tests. Direct comparisons between visits at each time point were made using paired samples *t*-tests or Wilcoxon signed-rank tests, depending on normality assessed by the Shapiro–Wilk test.

Reliability between visits for all derived metrics (bicarbonate and GI composite scores) was evaluated using typical error (TE), coefficient of variation (CV; with 95% confidence intervals), and intraclass correlation coefficients (ICC [1,2]; two-way random-effects, absolute agreement). ICC values were interpreted as follows: <0.50 = poor, 0.50–0.74 = moderate, 0.75–0.89 = good, and ≥0.90 = excellent reliability [30]. TE and CV were evaluated in the context of the variable's scale, with higher CV indicating greater relative variability.

For bicarbonate, a Monte Carlo simulation was performed to estimate the likelihood that an individual would exceed a +5 or +6 mmol·L^−1^ increase from baseline. For each 30-minute post-baseline time point, the change from baseline was assumed to follow a normal distribution based on pooled repeated measures. A total of 100,000 iterations per time point were generated to calculate the proportion of simulated values exceeding each threshold. An 80% probability threshold was predefined to indicate time points when the majority of individuals would likely meet these criteria. Prevalence of individual GI symptoms at each time point was summarised descriptively and compared between visits using chi-square tests.

All analyses were conducted in MATLAB R2025a (The MathWorks, Natick, MA, version 24.1.0) using base MATLAB for data input/output and interpolation, and the Statistics and Machine Learning Toolbox for mixed‐effects models (fitlme), repeated‐measures ANOVA (fitrm/ranova), nonparametric tests (ttest, signrank), χ² and distribution functions, and post-hoc contrasts (multcompare). Intraclass correlations were calculated with an open-source ICC.m routine [31], and Shapiro–Wilk normality tests via swtest.m [32]. Statistical significance was accepted at *p* ≤ 0.05 (two-tailed).

## 3 Results

Analysis of the 24-hour dietary recalls showed no significant differences in energy, macronutrient or sodium intake between visits. Mean ( ± SD) values for Visit 1 and Visit 2, respectively, were: energy, 2221 ± 640 kcal vs 2078 ± 636 kcal (*p* = 0.52); protein, 121 ± 44 g vs 114 ± 39 g (*p* = 0.54); carbohydrate, 272 ± 81 g vs 242 ± 81 g (*p* = 0.43); fat, 79.6 ± 27.8 g vs 79.4 ± 30.7 g (*p* = 0.98) and sodium, 1965 ± 1037 mg vs 1732 ± 1142 mg (*p* = 0.55). There were no violations of the 24-hour alcohol restriction, as confirmed via dietary recall. While most participants adhered to caffeine abstention, five reported consuming coffee the morning prior to the 24-hour period, with no subsequent intake; given typical caffeine metabolism, residual presence at the time of testing was unlikely.

Analysis revealed a significant main effect of time on blood bicarbonate concentration across the sampling period (*F*(1,204) = 20.91, *p* < 0.001), indicating a clear temporal pattern in the bicarbonate response. However, no significant main effect of visit (*F*(1,204) = 0.25, *p* = 0.615) or visit-by-time interaction (*F*(1,204) = 0.43, *p* = 0.512) was observed, suggesting that the time course of blood bicarbonate concentration was comparable across both trials.

**Figure 1.**
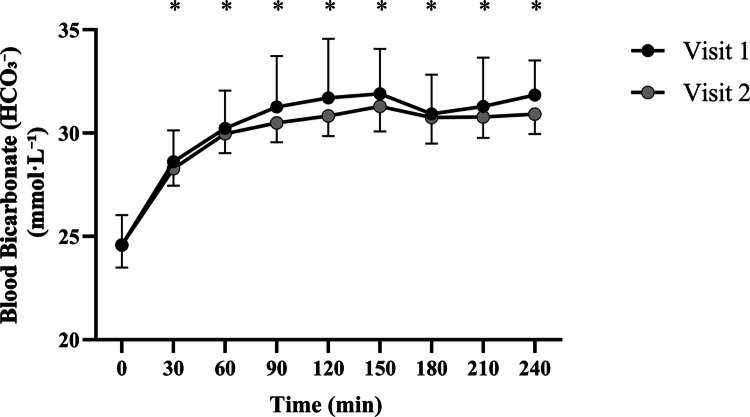
Time course of blood bicarbonate [HCO₃^−^] concentration following ingestion across two experimental visits. Data are presented as mean ± SD. Asterisks (*) denote time points significantly greater than baseline (0 min) within each visit (*p* < 0.05). No significant differences were observed between visits at any time point.

Repeated-measures ANOVA conducted within each visit confirmed a significant effect of time on blood bicarbonate levels in both Visit 1 (*F*(1,11) = 3301.4, *p* < 0.001) and Visit 2 (*F*(1,11) = 5234.0, *p* < 0.001) (See Figure 1). Post hoc comparisons revealed that in both visits, bicarbonate concentrations increased significantly above baseline from 30 minutes onwards. Specifically, relative to 0 minutes, significant increases were observed at 30, 60, 90, 120, 150, 180, 210, and 240 minutes in both visits (all *p* < 0.001). In contrast, no significant differences were detected between corresponding time points across visits (*p* > 0.05 at all time points), indicating high consistency in the temporal bicarbonate response between trials.

**Figure 2.**
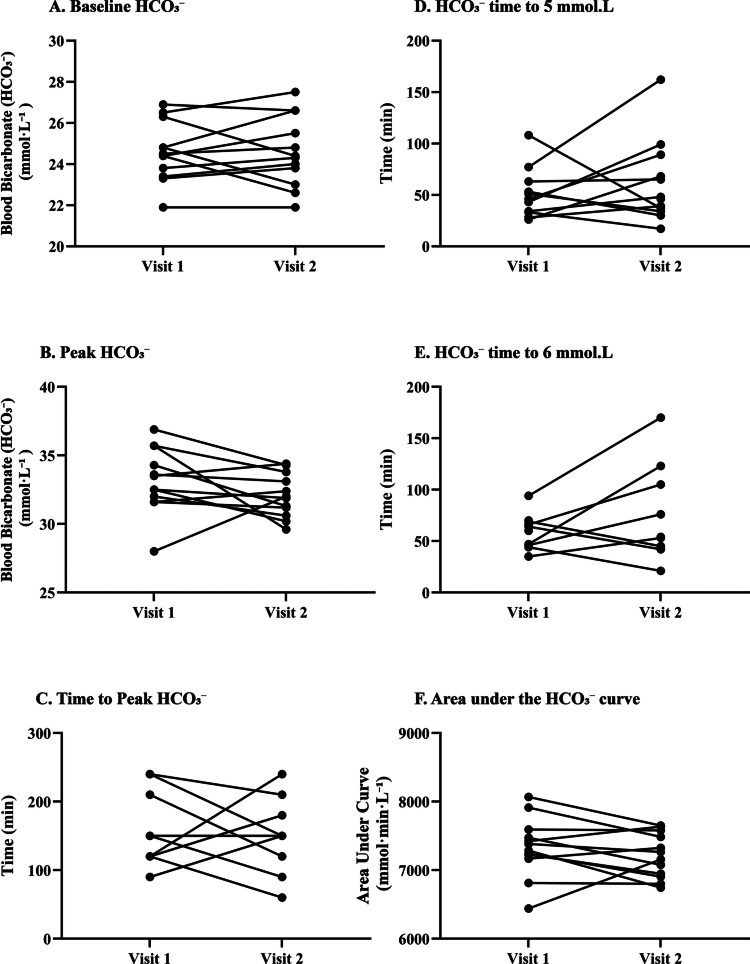
Individual variability in blood bicarbonate response metrics between Visit 1 and Visit 2. Panels show paired data for (A) baseline [HCO₃^−^], (B) peak [HCO₃^−^], (C) time to peak [HCO₃^−^], (D) time to reach + 5 mmol·L^−1^ increase, (E) time to reach + 6 mmol·L^−1^ increase, and (F) area under the [HCO₃^−^] curve (AUC). Each line represents one participant.

The reliability of individual blood bicarbonate response metrics between visits is summarised in Table 2 and illustrated in Figure 2A–F. Baseline [HCO₃^−^] showed moderate reliability (ICC = 0.72, 95% CI: 0.25–0.91), with a low typical error (TE: 0.87 mmol·L^−1^) and CV of 3.5%. The mean change between visits was negligible (0.0 mmol·L^−1^), and the absolute range of intra-individual differences was small (−1.9 to + 1.8 mmol·L^−1^), supporting its stability (Figure 2A). Peak [HCO₃^−^] demonstrated poor reliability (ICC = 0.23), despite a modest CV (5.4%) and TE of 1.8 mmol·L^−1^. Although group-level means were similar between visits, individual responses varied substantially (range: −6.1 to + 4.0 mmol·L^−1^; Figure 2B), indicating inconsistency in peak values within participants. TTP [HCO₃^−^] (Figure 2C) also exhibited poor reliability (ICC = 0.07), with a high TE (49.1 min) and CV of 32.5%. The mean difference between visits was small (2.5 min), but large individual variability was evident (range: −90 to + 120 min). For time to achieve a + 5 mmol·L^−1^ increase from baseline, one participant was excluded for not achieving the threshold in either visit. Among the remaining sample, reliability was poor (ICC = 0.19), with a TE of 30.8 min and a CV of 54.2%. The mean intra-individual difference was +11.5 min (range: −71 to +85 min; Figure 2D), suggesting limited within-subject consistency. Time to achieve a + 6 mmol·L^−1^ increase was analysed in only 8 participants (4 excluded for non-response in one or both visits). Again, reliability was poor (ICC = 0.36), with a TE of 29.6 min and a CV of 43.1%. Substantial variability in individual response timing was observed (mean difference: +21.3 min; range: −24 to +76 min; Figure 2E). In contrast, the area under the curve (AUC) showed moderate reliability (ICC = 0.56), with a low CV (3.5%) and TE of 254 mmol·min·L^−1^. The mean difference between visits was −121 mmol·min·L^−1^, with a range from −540 to +716 mmol·min·L^−1^ (Figure 2F), supporting its suitability as a more stable indicator of overall alkalotic exposure.

**Table 2.** Test–retest reliability of blood bicarbonate [HCO₃^−^] response variables across two experimental visits. Data are reported as mean ± SD for each visit, with corresponding *p*-values for paired comparisons, intraclass correlation coefficients (ICC [1,2]), typical error (TE), and coefficient of variation (CV) with 95% confidence intervals.

	Visit 1	Visit 2		ICC	TE	CV (%)
	Mean SD	Mean SD	*P* value	[95% CI]	[95% CI]	[95% CI]
Baseline [HCO₃^−^] (mmol·L^−1^)	24.6 ± 1.4	24.6 ± 1.6	1.0	0.72 [0.25, 0.91]	0.87 [0.6, 1.5]	3.5 [2.5, 6.0]
Peak [HCO₃^−^] (mmol·L^−1^)	33.2 ± 2.3	32.1 ± 1.5	0.16	0.23 [−0.29, 0.68]	1.8 [1.2, 3.0]	5.4 [3.8, 9.1]
TTP (min)	150.0 ± 50.5	152.5 ± 46.6	0.90	0.07 [−0.58, 0.62]	49.1 [34.8, 83.4]	32.5 [23.0, 55.1]
Time to 5^a^ (min)	51.1 ± 23.2	62.5 ± 39.7	0.40	0.19 [−0.44, 0.69]	30.8 [21.5, 54.0]	54.2 [37.9, 95.1]
Time to 6^b^ (min)	58.1 ± 17.7	79.4 ± 46.7	0.19	0.36 [−0.29, 0.82]	29.6 [19.6, 60.3]	43.1 [28.5, 87.7]
AUC (mmol·min·L^−1^)	7336.3 ± 417.8	7215.6 ± 312.3	0.27	0.56 [0.04, 0.85]	253.7 [179.7, 430.7]	3.5 [2.5, 5.9]

TTP = Time to peak; AUC = area under the curve;

a = 1 participant excluded due to achieving +5 mmol increase;

b = 4 participants excluded due to not achieving +6 mmol increase.

The probability of achieving an absolute increase in blood bicarbonate of +5 or +6 mmol·L^−1^ from baseline at each time point is presented in Table 3. Based on the predefined threshold of 80% probability, values indicative of a high likelihood of response were observed from 120 minutes onwards for the +5 mmol·L^−1^ criterion (≥83.9% from 120–240 min), and from 150 minutes onwards for the + 6 mmol·L^−1^ threshold (≥69.7%). At 30 minutes post-ingestion, the likelihood of achieving either threshold was low (18.2% and 4.3% for +5 and +6 mmol·L^−1^, respectively), but this increased progressively over time. By 90 minutes, 78.8% of individuals were predicted to exceed +5 mmol·L^−1^, approaching the decision threshold, while only 57.1% surpassed +6 mmol·L^−1^. The peak probability for +5 mmol·L^−1^ was 85.8% at 240 minutes, while the highest probability for +6 mmol·L^−1^ was 69.7% at 150 minutes, indicating that the majority of individuals are likely to attain this higher threshold, although a sizeable minority may not respond even several hours post-ingestion.

**Table 3.** The probability of individuals exceeding + 5 mmol·L^−1^ and + 6 mmol·L^−1^ increases in blood bicarbonate [HCO₃^−^] concentration at each time point post-ingestion.

Time post ingestion (min)	Probability of Increases above 5 mmol·L^−1^ (%)	Probability of Increases above 5 mmol·L^−1^ (%)
30	18.2	4.3
60	62.8	38.1
90	78.8	57.1
120	83.9	65.5
150	84.4	69.7
180	83.5	58.1
210	80.1	60.5
240	85.8	68.3

Probability values estimated with Monte Carlo simulation *(n = 100,000).*

**Figure 3.**
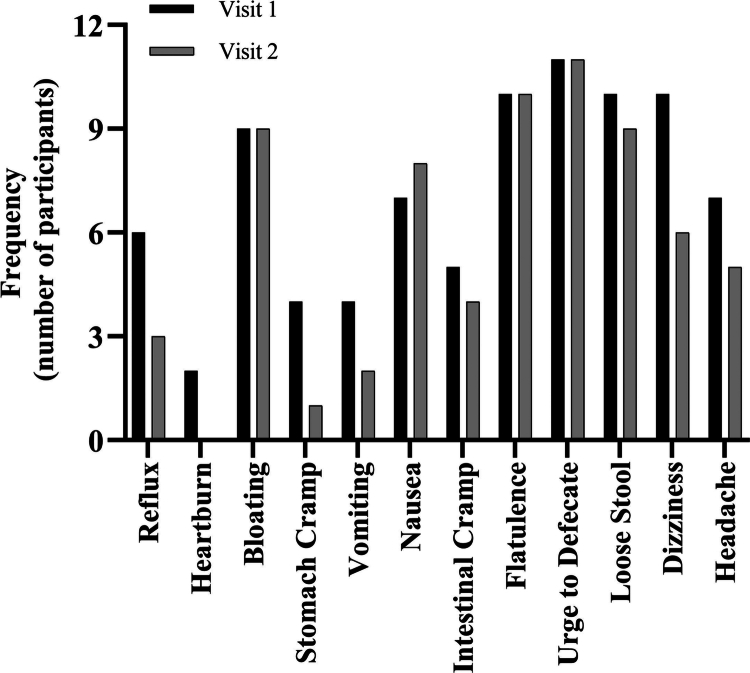
Frequency of gastrointestinal symptoms reported at Visit 1 and Visit 2. Bars represent the number of participants reporting each symptom at any time point during the 240-minute post-ingestion period.

All 12 participants reported at least one gastrointestinal (GI) symptom at Visit 1, compared with 11 participants at Visit 2. As shown in Figure 3, the most frequently reported symptoms during both visits were urge to defecate (92%), flatulence (83%), and bloating (75%). Symptom frequencies were comparable across visits, with no significant differences observed for any individual symptom (*p* > 0.05).

**Figure 4.**
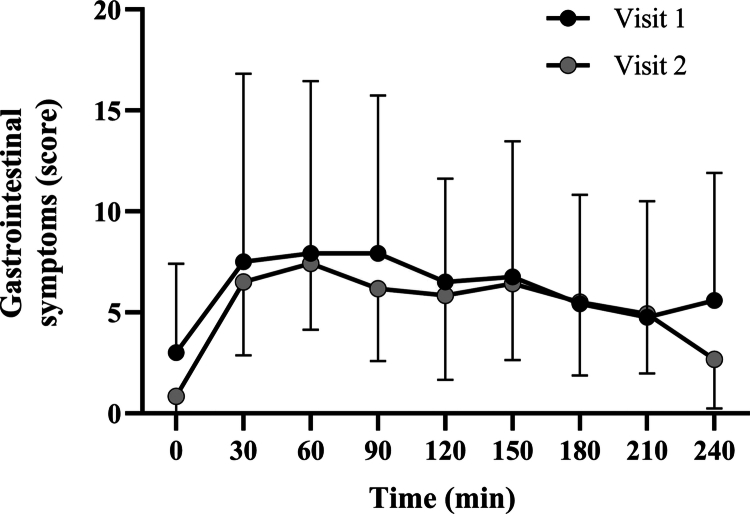
Mean (±SD) composite gastrointestinal symptom scores over time for Visit 1 and Visit 2. Scores represent the sum of corrected ratings across 12 individual symptoms at each time point (range: 0–108).

Composite GI scores (range 0–108) were derived by summing corrected symptom ratings at each time point. No significant main effect of Visit was detected (F(1,211) = 0.90, *p* = 0.34), nor was there an effect of Time (F(1,211) = 0.08, *p* = 0.78), or a Visit × Time interaction (F(1,211) = 0.04, *p* = 0.85), indicating that the overall GI symptom burden was consistent between visits and did not change meaningfully over time. Summary statistics for composite scores by visit and time point are presented in Figure 4.

Overall GI discomfort, defined as the sum of composite scores across all time points, did not differ significantly between visits (*p* = 0.38). Intertrial reliability analysis showed a typical error (TE) of 2.63 (95% CI: 1.86–4.46) and a coefficient of variation (CV) of 46.63% (95% CI: 33.03%–79.17%). The intraclass correlation coefficient (ICC [1,2]) was 0.61 (95% CI: 0.10–0.87), F(11.0, 11.9) = 4.11, *p* = 0.01, reflecting moderate reliability for overall composite GI scores across repeated trials.

## 4 Discussion

The primary aim of this study was to examine the intra-individual reliability of blood bicarbonate responses and GI symptoms following SC supplementation. Our findings demonstrate that SC ingestion produced significant and sustained increases in blood [HCO₃^−^] over time, with a consistent temporal profile across visits. While group-level kinetics were reproducible, reliability analyses revealed individual-level variation. Baseline [HCO₃^−^] and AUC demonstrated moderate test–retest reliability, whereas peak concentrations and all time-based metrics exhibited poor reproducibility. Furthermore, although most participants exceeded the + 5 mmol·L^−1^ threshold, fewer consistently achieved above + 6 mmol·L^−1^, underscoring inter-individual variability in alkalotic response. Composite GI symptom scores did not differ significantly between visits or over time, with moderate reliability observed for overall discomfort.

### 4.1 Blood bicarbonate kinetics, reliability and thresholds

Sodium citrate ingestion in our study elicited a rapid rise in blood [HCO₃^−^] within 30–60 min, followed by a plateau that persisted through 240 min (end of observation). This temporal profile aligns with previous findings and mirrors the delayed-but-sustained alkalosis seen with sodium bicarbonate, albeit with some differences in absorption kinetics and GI tolerability [18,20]. Notably, the earliest significant increase above baseline occurred by 30 min, and peak values were typically reached around 150–180 min. This timing is consistent with prior reports of time-to-peak ranging roughly from ~2.5 hours up to ~4–5 hours post-ingestion under various protocols [18,33,34]. Protocol features such as dose (0.4–0.5 g·kg^−1^), formulation, and ingestion duration appear to modestly shape the kinetics rather than redefine them. Across studies, the group mean TTP commonly falls within ~180–240 min, with individual values extending towards ~300 min [18,20,34,35]. In capsules at 0.4–0.5 g·kg^−1^, [HCO₃^−^] typically rises by ~5–6 mmol·L^−1^ and remains elevated for several hours [34], while delayed-release formats have reported earlier group means (≈145 ± 28 min), indicating that formulation can shift TTP by tens of minutes rather than hours [33]. Solution-based protocols yield peaks around ~180–212 min, broadly overlapping with capsule data [18]. Extending ingestion duration from ~15 to ~60 min may slightly delay TTP, again on the order of tens of minutes [35]. In head-to-head comparisons, the magnitude of alkalosis with sodium citrate is comparable to sodium bicarbonate at recommended doses, but citrate tends to attain ≥+6 mmol·L^−1^ later, typically nearer ~3 h versus ~2 h for bicarbonate [20]. These comparative kinetics underscore the practical recommendation to ingest sodium citrate at least ~3 hours before exercise to ensure peak alkalosis is attained [18], while also potentially benefiting from its lower incidence of acute GI symptoms relative to bicarbonate [33].

Despite the reproducibility of group-level responses, substantial inter-individual variability in the timing of peak [HCO₃^−^] was evident. TTP exhibited poor reliability, with wide intra-individual differences observed between visits (range: −90 to +120 min; see Figure 2C). This suggests that fixed sampling windows may not accurately capture maximal alkalosis for all individuals and has clear implications for applied practice and reinforces the need for individualised protocols. Without personalised profiling, athletes may mistime ingestion relative to performance, thereby failing to optimise buffering capacity at the point of exertion. Baseline [HCO₃^−^] and AUC both demonstrated moderate reliability (CV = 3.5%) and acceptable typical error values. These findings suggest that such metrics may offer suitable endpoints for monitoring systemic alkalosis in repeated testing scenarios, provided that standardised pre-analytical controls are in place. Importantly, AUC reflects the integrated exposure to elevated [HCO₃^−^] across the entire observation period, capturing the duration and consistency of the alkalotic state. For exercise events lasting several minutes, this cumulative exposure may be more relevant than transient peak concentrations when considering ergogenic potential. In contrast, peak [HCO₃^−^] concentration and all time-dependent measures (e.g. TTP, time to +5 mmol·L^−1^, time to +6 mmol·L^−1^) exhibited ICCs between 0.07-0.36, with wide typical errors and CVs often exceeding 40%, indicating that these measures were highly variable between repeated exposures. The poor reproducibility of these timing variables likely reflects the influence of individual gastrointestinal transit, absorption kinetics, and potential biological noise in bicarbonate regulation.

Practically, these findings caution against using fixed ingestion-to-peak intervals across individuals. While the general time course of alkalosis is predictable at the group level, reliance on specific time-to-threshold or peak values to inform performance timing may lead to mistimed buffering windows. Instead, repeated within-subject testing may be required to optimise supplementation schedules for athletes seeking to maximise the ergogenic potential of SC.

Monte Carlo simulations showed that most individuals were likely to exceed a +5 mmol·L^−1^ [HCO₃^−^] increase from 90 minutes onward, with the probability >80% between 120 to 240 minutes. In contrast, the likelihood of achieving a +6 mmol·L^−1^ rise peaked at 69.7%, falling below the pre-defined threshold of high certainty, indicating that a substantial minority may not reach this higher benchmark, even at later time points. These findings support a +5 mmol·L^−1^ increase as a realistic benchmark for ergogenic benefit [14], with current evidence suggesting no consistent performance advantage below this [36]. This simulation-based approach moves beyond static group means, providing a nuanced, probability-based perspective for practitioners. These data support the adoption of a 2–3-hour ingestion window for SC to maximise the likelihood of achieving sufficient systemic alkalosis, particularly for events where acid–base buffering capacity is a performance-limiting factor.

### 4.2 Gastrointestinal discomfort

Gastrointestinal discomfort is a well-documented limitation of alkalinising supplements. Results showed that overall GI symptom burden was moderate and stable across trials, with the most common symptoms (urge to defecate, flatulence, bloating), consistent with prior reports of induced GI effects following SC ingested in a solution (16) and observed in over 75% of participants in both trials. Comparative work using 0.4 g·kg^−1^ SC in capsule form has reported minimal GI burden for many participants, with alkalosis preserved despite lower dose and different delivery [34]. Where SC has been contrasted directly with sodium bicarbonate, overall GI symptom severity appears broadly similar between supplements, although the peak in symptoms tends to occur later with SC, mirroring its delayed absorption profile [20]. Our higher dose in solution and the hypertonic nature of the drink likely contributed to the greater frequency of mild-to-moderate symptoms observed here, highlighting the relevance of formulation, dilution, and ingestion tempo. Practical strategies to mitigate GI symptoms include splitting the dose into two ingestion points (e.g. 0 and 30 min) [35], using capsules over solutions [17], co-ingesting SC with a small carbohydrate-containing snack [20], and aligning ingestion with habitual pre-event meals [15]. These strategies have demonstrated improved tolerability in recent protocols without compromising the alkalotic response, and warrant further exploration in performance-based settings.

Reliability analysis of composite GI scores revealed only moderate agreement between visits (ICC = 0.61, CV = 46.6%). This suggests that although the average symptom profile remained stable across trials, individual experiences varied substantially. In practical terms, some participants reported minimal or no discomfort in both sessions, while others experienced marked fluctuations in symptom severity, complicating efforts to predict GI tolerance based on a single exposure. These findings underscore the importance of individual trialling before competition use, particularly when higher doses are employed or when SC is consumed alongside other ergogenic supplements or meals. While the 0.5 g·kg^−1^ dose used in the present study was chosen to balance efficacy and tolerability, the inter-individual variability in symptom response suggests that even this moderate dose may be poorly tolerated by some athletes. Future studies should explore individual thresholds for GI side effects and consider strategies such as dose splitting or alternative formulations to improve tolerability.

### 4.3 Practical applications and future considerations

Given the group-level consistency but individual variability in response timing, practitioners should avoid relying on fixed ingestion-to-performance intervals. For athletes aiming to align peak alkalosis with exercise onset, repeated profiling may be necessary to identify their optimal ingestion window. Concentration-based metrics (baseline [HCO₃^−^] and AUC) showed superior reliability and may be better suited to tracking an athlete’s readiness or responsiveness to supplementation. These indices are less influenced by transient fluctuations in absorption or sampling timing and offer a more stable reflection of total buffering exposure. Our data support a 2–3-hour ingestion window to maximise the likelihood of achieving ergogenic alkalosis, though individual profiling remains essential.

Several strengths and limitations of this study warrant discussion. The use of repeated, tightly controlled laboratory trials with standardised dietary intake, exercise avoidance, and identical SC dosing across sessions enhances the internal validity of the reliability analyses. However, one limitation is that water intake during the 4-hour observation window was allowed ad libitum and not quantified. Uncontrolled hydration may have introduced minor variability in gastric emptying or dilution of blood bicarbonate. Future studies should consider standardising fluid intake or tracking it to determine its influence on bicarbonate kinetics and gastrointestinal responses.

The inclusion of interpolation (pchip) and continuous kinetic modelling allowed for robust characterisation of individual response trajectories across the 4-hour post-ingestion window. The blood sampling protocol (nine capillary samples over 240-minutes) was designed to provide a sufficiently detailed kinetic profile without introducing undue participant burden or compromising sampling quality.

While the iSTAT device has previously demonstrated acceptable reliability for resting blood [HCO₃^−^] assessment, capillary measurements are inherently more variable than venous sampling, and future studies may consider parallel sampling approaches for validation. Despite the strengths of repeated-measures design and detailed statistical modelling, the sample was limited to healthy, recreationally active males. This lack of female representation restricts the generalisability of our findings. Sex-based physiological differences; including body composition, blood volume, and hormonal fluctuations, could influence buffering responses to alkalotic supplements. For instance, the menstrual cycle or oral contraceptive use might alter baseline electrolyte balance or renal handling of bicarbonate and could modulate GI motility [37,38]. Future research should therefore include female athletes and examine whether sodium citrate’s kinetic and symptomatic profile differs by sex. We specifically recommend investigating females across different menstrual cycle phases to determine if hormonally driven variations (e.g. in luteal vs follicular phase) affect the magnitude or reliability of the [HCO₃^−^] response and tolerance to sodium citrate.

Additionally, although 12 participants were initially enroled, certain bicarbonate metrics (notably time to +6 mmol·L^−1^) suffered from incomplete data due to participants not achieving the specified thresholds. While this reflects real-world variation in responsiveness, it reduces statistical power for reliability estimates in these cases. Additionally, although our 4-hour post-ingestion sampling period was based on prior literature, it may not have been sufficient for all individuals. Participants who had not reached a definitive peak [HCO₃^−^] by 240 min were effectively right-censored in our data (i.e. their true time to peak could be beyond the observation window). This constraint likely inflated the variability in measured TTP and underpins the poor reliability we found for that metric. Future studies should consider extending the sampling duration beyond 240 min.

We also acknowledge that the absence of a non-supplemented control means we did not measure baseline day-to-day fluctuations in [HCO₃^−^] or GI symptoms in the absence of SC. However, extensive prior research has demonstrated that sodium citrate ingestion (0.5 g·kg^−1^) induces significantly greater alkalosis than no supplement [34]. Thus, while a placebo was beyond the scope here, future studies aimed at performance effects should include a control condition to fully isolate the supplement’s impact. Furthermore, participants were informed of potential GI effects, and expectancy or anticipatory responses were not measured, which could have influenced subjective symptom reporting across visits. Future work should quantify expectancy and prior supplement exposure to delineate physiological from expectation-driven GI responses. Finally, the GI discomfort scale, though validated, relies on subjective self-report and may be influenced by individual perception and symptom sensitivity. Objective markers of GI motility or transit may complement such tools in future work.

Future research should explore the intra-individual stability of bicarbonate kinetics in diverse populations (e.g. female athletes, elite performers), evaluate the utility of multiple time-point sampling in applied environments, and investigate potential moderators of response such as gut microbiota, genetic variability, and co-ingested nutrients. Moreover, the ergogenic implications of the observed variability should be directly tested using performance outcomes tied to individually determined [HCO₃^−^] response profiles.

## 5 Conclusion

This study provides novel insight into the intra-individual reproducibility of blood bicarbonate responses following sodium citrate supplementation. While baseline [HCO₃^−^] and overall alkalotic exposure (AUC) demonstrated moderate reliability, metrics critical for timing ingestion, namely time to peak and time to exceed ergogenic thresholds, were inconsistent within individuals. These findings reinforce that while group-mean responses may appear stable, individual trajectories often diverge, which has clear implications for personalised supplementation strategies.

## Appendix

### Appendix 1. Gastrointestinal symptom severity questionnaire

Participants rated the severity of each symptom using a 10-point Likert scale, where 0 = “no problem at all” and 9 = “the worst it has ever been”.

**Table:**

Symptom	0	1	2	3	4	5	6	7	8	9
Reflux (acid backflow)										
Heartburn (burning chest pain)										
Bloating (full, tight abdomen)										
Upper Abdominal Cramp (tight pain)										
Vomiting (expelling stomach contents)										
Nausea (feeling of wanting to vomit)										
Intestinal Cramp (tight pain)										
Flatulence (gas expulsion)										
Urge to Defecate (bowel movement urge)										
Loose Stool (watery faeces)										
Dizziness (light-headedness)										
Headache (pain in head)										
