# Correction

**DOI:** 10.1080/15502783.2026.2633020

**Published:** 2026-02-16

**Article title**: Intra-Individual Reliability of Blood Bicarbonate Responses and Gastrointestinal Symptoms Following Sodium Citrate Supplementation

**Authors**: McManus, C. J., Liew, B. X. W., Waterworth, S. *P*. W., & Chung, H. C.

**Journal**: *Journal of the International Society of Sports Nutrition*

**Bibliometrics:** Volume 23, Number 01, pages 1−14

**DOI:** http://doi.org/10.1080/15502783.2026.2629830

The article was published with Box Head error in Table 3 on page 8:

Probability of Increases above 5 mmol·L^−1^ (%)

Correct Box Head is:

Probability of Increases above 6 mmol·L^−1^ (%)

The article has been republished with the errors corrected.

